# Researches on the Possibility of Temporarily Reanimating Persons Dying of Disease

**Published:** 1859-04

**Authors:** E. Brown-Sequard


### TRANSLATIONS FROM FOREIGN JOURNALS.

### RESEARCHES ON THE POSSIBILITY OF TEMPORARILY REANIMATING PERSONS DYING OF DISEASE.

BY DR. E. BROWN-SEQUARD.

Translated by THOMAS BEVAN, M.D., from the Journal de Physiologie for Oct., 1858.

For a number of years my researches on the transfusion of blood commenced in 1846, and continued almost without interruption to the present, have given me such results as demonstrated positively, that life could be re-established for a certain time in the mammalia dying of various diseases, and especially peritonitis.

I will only report here some few of the principal facts that I have observed, proposing at a future time to publish them in a more extended memoir on the transfusion of blood.

The experiments which have succeeded, were made upon animals that had ceased to breathe for variable periods (in one case 17 minutes), and in which all trace of voluntary movements and sensibility had disappeared. As to the heart, in the majority of the cases, the sounds were still heard, but the pulse had ceased in the arterial trunks of the extremities, and often even in the carotid; in two cases the beating of the heart appeared to have ceased for several minutes, the last convulsive agony had passed, and the pupil was dilating, or already dilated.

In a word, death was imminent, and no one assuredly could have supposed a spontaneous return to life possible.

The diseases of which these animals died that I am now describing, were for the most part inflammations of the peritoneum and pleura, consecutive to wounds of these membranes, made in experiments on different abdominal viscera and the par vagum nerves, sympathetic or diaphragmatic in the thoracic cavity. In several cases death was due to the extirpation of the suprarenal capsules of the kidneys.

In all these cases, without exception, asphyxia was the immediate cause of death. This is too important a question to be treated incidentally; I shall make it the subject of a series of memoirs on the causes of death in the acute diseases of men and animals. But I ought to say, for the present, that asphyxia in a great number of cases of lesions of the abdominal viscera, in the experiments which were the object of the present work, were, in great part, the consequence of enfeebled movements of the heart, the *primitive* cause of which (not the only cause) was the irritation of the ramifications of the great sympathetic nerve in the abdomen.

Whatever was for the rest the mode of production of the asphyxia, this existed, and, in endeavoring to establish life for a certain time in dying animals, it ought consequently to be taken into account. I was thus conducted to try if pulmonary insufflation would have any influence. In eight or ten cases of dying animals, it had a slight effect, in augmenting the force of the movements of the heart, and one only appeared to become sensible during a few minutes. It is entirely different when employed before the agony, but, as that is not the subject of this article, I will limit myself to stating, that the death agony may be retarded one or two hours by insufflation.

This artificial respiration being insufficient to recall life in animals dying of febrile maladies, I thought if, by employing other means, I could succeed any better, I ought to say to those persons who imagine galvanism to be a useful means in re-establishing life in the asphyxiated, that I have not employed it in a single case, because I knew that the best means of extinguishing the remains of life in the dying individual, was to submit their nerves and muscles to the exhausting excitative action of galvanism.—(See *Lois des phenomenes dynamiques de l'economie animale*, No. 1, p. 7–10 *de ce Journal*). I thought of the employment of transfusion of blood, not with the foolish idea of the first transfusors, who believed they could cure grave diseases by substituting fresh blood for the diseased fluid, but with the desire to see the influence of this operation on the scarcely living organism, necessarily condemned to death.

I practiced the operation, sometimes one way and sometimes another, but space being wanting here, I will limit myself, apropos of a case that I now report, to describe the method which has succeeded with me best, and add afterwards a summary indication of some modification of this method.

Exp.—In October, 1851, a dog, in whom I had cut the great sympathetic nerve of the abdomen, was attacked with peritonitis, and after two or three days' sickness, presented the signs of approaching death known to all virisectors. The voluntary movements had already ceased for sometime (I did not note how long), sensibility and reflex movements had everywhere disappeared, even in the eyes; the last respiratory movements, those of the jaws and nostrils, had occurred, and were arrested. The convulsive agony (very feeble in this case) in the members, the face and the eyes, no longer existed, except in a tremor limited to a very small number of muscles. The animal had expelled the contents of the bowels and the urine, the pupil was dilated, and the beating of the heart was no longer felt; the only sign of life which yet persisted, consisted in a vague sound which no longer resembled the heart's action, was heard about eight times in the twelve seconds immediately preceding the transfusion of blood from another dog, made in the following manner: a silver tube, having the form of a T, was fixed by the branch which corresponds to the chapter of the T in the interior of the right carotid of the dying dog, and the free extremity a little curved of the other branch was introduced, and fixed in the left carotid of another dog, whose head and body were firmly attached to a table. Immediately the arterial blood of the healthy dog circulated in the two opposite directions, towards the head and heart, in the carotid of the dying animal.

At the same time the left jugular vein and one of the femoral veins was opened in the dying dog. These two veins at first gave no blood, but the jugular soon, and the femoral twenty or thirty seconds after, commenced to give a little. During the first fifteen seconds, by pressing on the carotid, from which the transfused blood came, I diminished the quantity of this liquid, and after two minutes the operation was ended, and ligatures put on the carotid of the living dog. The jugular vein was left open four or five minutes, during which the beating of the heart commenced to be felt. As fast and proportionally as the blood flowed from the jugular the pulse re-established itself. I then had recourse to insufflation; but, as I only had one assistant, the trouble of the operation prevented my counting the pulse during the first twelve minutes. When it was counted it was very feeble, and beat sixty-four times per minute. Insufflation was continued almost without interruption for half an hour. The cornea was found to be sensitive after the eighth minute of the insufflation. A few minutes later, respiratory efforts showed themselves. Lastly, after twenty minutes, the animal made voluntary movements, and, when insufflation was ceased, respiration occurred very rapidly, but without much force. The return to life, however, was complete as to the existence of all the principal functions of animal and organic life. The animal, although feeble, raised himself up on his fore paws, and wagged his tail when caressed. The pulse, small and feeble, was rapid, (110 to 120 then; several hours after, 80). At last, after remaining four or five hours in this condition, the animal sank anew and died (I was going to say re-died), eleven hours and a half after the transfusion.

I will add a few remarks on the details of this experiment. I did not weigh the dogs submitted to the experiment, and I am ignorant of the quantity of blood transfused, and of what quantity was lost by the dying dog; but I noted that the animal furnishing the blood was small, and that the other was large, so that, taking account of these facts, and the duration of the transfusion, with several other circumstances, I have reason to believe that the dying animal did not receive more blood than he lost. However that may be, subsequent experiments have shown me that the moribund animal received more blood than he ought, and that it is probable the return to life would have been more rapid if the quantity had been less.

There are in this experiment several causes of the return to life: 1, the passage of the healthy arterial blood in the coronary arteries gave to the muscular fibres of the heart more irritability, and, in consequence, rendered them more capable of obeying the excitations, whatever they may have been, which caused their rythmic contractions; 2, the passage of the healthy arterial blood in the arteries of the encephalon; 3, the substitution of healthy blood for the blood changed by inflammatory disease, and by the asphyxia which exists in the agony; 4, pulmonary insufflation; 5, disgorgement of the right side of the heart, by the bleeding from the jugular.

Of these five causes of temporary return to life, there is one, the insufflation, of which I have said it was incapable alone, of producing this result. As to the others, I ought to say, they are nearly as inefficacious as the insufflation, when employed alone. Thus, the injection of the red blood towards the heart, by the carodid in dying animals, only succeeds in augmenting for a time the force and rapidity of the movements of this organ, but without re-establishing the circulation. Again, the transfusion of blood by the vein only hastens the complete cessation of the movements of the heart, if made without the right side of the heart being able, by the jugular bleeding, to disembarrass itself of the fulness which prevented its contraction in the asphyxia. The same, also the transfusion by the carotid towards the encephalon, only adds to the difficulty of the contractions of the right side of the heart, so that the opening of the jugular alone renders the heart more capable of acting, but only for a very short time.

M. Segolas, a long time ago,* and after him, J. Reid,† Dr. H. Lonsdale,‡ Dr. Cormack,|| and, lastly, Dr. Struthers,§ have

---

* Journal de la Physiologie de Magendie, vol. iv., 1824, p. 290.

† Physiolog. Anat. and Pathol. Researches, 1848, p. 50–60.

‡ Edinb. Med. and Surg. Journal, 1836, No. 138.

|| Treatise on Creosote, 1839, p. 84.

§ Edinb. Journal, Nov., 1856, p. 241–56.

established in the asphyxia of hanging, that following blows upon the head, or in certain cases of poisoning, that the movements of the heart may be increased, or reproduced, if arrested by bleeding from the jugular vein, by removing by this means the distension of the right cavities of the heart. This excellent means employed alone in the dying animal, where the disease is inflammatory, is absolutely powerless. Even if joined with pulmonary insufflation, I have never succeeded in re-establishing the circulation, and yet less the respiration and the functions of animal life. The only result of the simultaneous employment of these two means has consisted in an augmentation of the movements of the heart. But in those cases where I have employed them, even in the commencement of the agony, or before it was manifested by convulsive movements, I have seen quite often the circulation and respiration re-established during one or two hours, and sometimes even a momentary return of the functions of animal life.

I tried in a number of cases, transfusion by the carotid in the two opposite directions, taking care that the jugular was open, during and a little after the transfusion, and without making insufflation. The return to life was more rare in this class of cases than where insufflation was used, but it took place in several animals which survived two or three hours. I do not know the real proportion of animals reanimated for a time, in the number of trials I made, nor what was the average of this new life of the animals upon which I practiced the transfusion, insufflation and jugular bleeding, because I have not by me all the records of my experiments, but, according to the notes I have before me, in eleven experiments made on dogs, cats, and adult rabbits, nearly all dying of peritonitis, I see that, in four individuals reanimation was complete during two, three and four hours (one dog, one cat and two rabbits), and that three others recovered for one or two hours the circulation, respiration and the reflex faculty, without voluntary motion or sensibility having reappeared, while in four others no result was had, except a slight augmentation of the movements of the heart.

Of late years, having learned from numerous researches on transfusion, that the blood need not be either warm, or possess its fibrine,* I have renounced the employment of the healthy animal to make the transfusion. After having bled from the jugular and commenced insufflation, I inject at different times, and very slowly, a quantity of blood, a little less than that the animal has lost, I make the injection alternately towards the head and towards the heart, so as to act upon the encephalon, with the view to re-establish respiration, and on the muscular fibres of the heart, to augment their irritability.

Can we draw any conclusions from these experiments, relative to the combined employment of transfusion, insufflation and the jugular bleeding, in men dying of inflammatory or other diseases ? It is evident that in the immense majority of cases, it would be useless, if not cruel, to snatch from death for a necessarily short time, an individual of our race, where irreparable material lesions condemned him to death. But there might be cases presented in which it would be important that the intelligence, speech, the senses and voluntary movements should be rendered to the dying. Now, the facts mentioned in this paper, in demonstrating that *all the functions of animal life may be re-established for several hours, in animals in whom the agony has already almost given place to death*, render it extremely probable that the intellectual faculties, the senses, speech, etc., might be re-established for a few hours in individuals who have lost these faculties, and in whom the death agony has commenced.

The success of these various operations would be the more probable, as to practice them we should not wait, as I have done in animals, until the death agony had made considerable progress; and much less, until it had almost entirely ceased.

---

* I have recently found, however, that the defibrinated blood presents thi danger, that in mingling itself with the blood of the animal, into which it is transfused, it sometimes causes its sudden coagulation, but it is probable that, in using a thousandth or fifteen hundredth part of the caustic ammonia to the defebrinated blood, one avoids the only danger known to me in its usage.—See the second part of the analysis of the book of Dr. Richardson, in the present number of this journal.

